# Outcomes of Pregnancy-Associated Breast Cancer: A Tertiary Care Cancer Hospital Experience

**DOI:** 10.7759/cureus.78162

**Published:** 2025-01-28

**Authors:** Muhammad Waheed, Sameen Bin Naeem, Maryam Imran, Fareeha Fatima, Shahida Parveen, Neelam Siddiqui, Tahira Yasmeen

**Affiliations:** 1 Medical Oncology, Shaukat Khanum Memorial Cancer Hospital and Research Centre, Lahore, PAK; 2 Rheumatology, Foundation University School of Health Sciences, Rawalpindi, PAK

**Keywords:** breast, cancer, neoadjuvant, outcomes, pregnancy

## Abstract

Introduction

Pregnancy-associated breast cancer (PABC) is defined as breast cancer diagnosed during pregnancy or within one year postpartum. There is limited data on this, especially from low- and middle-income countries (LMICs), warranting further exploration. Factors such as restricted healthcare access and resources and cultural beliefs that influence awareness are the main barriers to LMICs.

Study design

This retrospective study included 44 adult patients aged 30 years and older. Only patients who received treatment and subsequent follow-up at our institution were included. The study was conducted at the Department of Medical Oncology, Shaukat Khanum Memorial Cancer Hospital and Research Centre, Lahore. The data were extracted from the hospital records over a 10-year period from 2010 to 2020.

Patients and methods

A total of 44 patients were included, with a median age of 33 years. Eleven (25%) had triple-negative disease, 23 (52.3%) were hormone receptor (HR) positive and HER2 negative, three (6.8%) were HER2 positive and HR negative, and seven (15.9%) had triple-positive disease. A total of 81.8% presented with early-stage breast cancer (EBC) and received either neoadjuvant (n = 32) or adjuvant (n = 11) chemotherapy and surgery. Trastuzumab, tamoxifen, and radiotherapy were administered post-delivery. Factors associated with mortality were analyzed using chi-square statistics and the Mann-Whitney U test. Survival analysis for overall survival and disease-free survival was performed using Kaplan-Meier survival analysis.

Results

At a median follow-up of 27 months, the estimated three-year event-free survival for EBC and locally advanced breast cancer was 82% (95% CI: 65.2-100) and 56% (95% CI: 42-75.6%), respectively, and 24% (95% CI: 10.1%-58.5%) for metastatic breast cancer. Of the 44 patients, 14 had terminations, whereas 29 had full-term deliveries (FTDs). Patients with PABC showed a 92.6-month mean survival, but tumor recurrence significantly impacted outcomes, with a 61.1% mortality rate and a reduced median survival of 44.5 months compared to 62.5 months in non-recurrent cases. Recurrence emerged as the strongest predictor of mortality.

Conclusion

Future research should improve PABC patient outcomes by improving diagnostic methods, refining treatment protocols, investigating long-term effects, developing early detection tools, tailoring treatment plans, and evaluating treatment impact on fetal health. It is essential to establish guidelines for mother and child safety and address emotional and psychological needs.

## Introduction

In 2020, approximately 19.2 million new cancer cases and 10 million cancer deaths were reported worldwide. Breast cancer is the leading cause of cancer deaths among women globally, representing one in four cancer diagnoses and one in six cancer deaths overall [1]. In Pakistan, there were an estimated 26,000 new cases of breast cancer, accounting for 14.5% of total cancer patients, and is the number one cause of cancer-related mortality [1].

Pregnancy-associated breast cancer (PABC) is rare and is associated with poor outcomes [2]. The term "pregnancy-associated breast cancer" refers to breast cancer that is detected either during pregnancy (BCP) or within the first year after childbirth (BCPP).

Although PABC includes a variety of subtypes of breast carcinomas, invasive ductal carcinomas with a high tumor grade and large tumor size are the main characteristics of PABC. Given the increased probability of lymph node involvement, these tumors are frequently detected at a later stage [3]. Furthermore, hormone-negative tumors (triple-negative or HER2-amplified tumors) and high Ki-67 proliferation rates are features that most PABCs have [4].

PABC differs significantly from non-pregnant patients in terms of clinical presentation, diagnosis, and treatment [5]. The use of diagnostic modalities to successfully identify breast cancer in pregnant and lactating females is limited. The increased breast density in these patients may reduce the sensitivity of imaging methods, particularly mammography. Other imaging tests required for accurate staging are avoided because of the potential harm that radiation and contrast media can cause to the fetus. Even biopsies of suspicious masses are done cautiously when necessary because the formation of milk fistulas may complicate the procedure [6,7].

Pregnancy imposes significant restrictions on the use of some effective treatment options due to potential teratogenic effects on the fetus. Systemic therapies, such as chemotherapy, hormonal therapy, and immunotherapy, can be particularly problematic. Therefore, treatment for PABC requires careful design and may differ significantly from regimens used for the general population.

Pakistan's maternal and infant mortality rates are higher than those of developed nations due to several factors, including inadequate access to and distribution of healthcare facilities, sociocultural norms, and a lack of funding for women's health [8]. It is a difficult task to treat a pregnant patient with cancer while placing equal emphasis on the health of the fetus. In addition, delayed diagnosis is caused by a lack of knowledge about cancer and a lack of resources and expertise. Thus, the purpose of this study was to evaluate the clinicopathological and prognostic factors for PABC affecting survival in our population by analyzing the PABC cases from the registry created at the Shaukat Khanum Hospital and comparing them with published literature.

## Materials and methods

This research is a retrospective cohort study carried out at Shaukat Khanum Memorial Cancer Hospital in Lahore, Pakistan. The data of patients diagnosed with PABC were extracted from the hospital records over a 10-year period from 2010 to 2020. The extracted data were anonymized after removing the individual patient identifiers, e.g., name and hospital record number. The study was approved by the hospital's research ethics review committee (IRB-EX-05-09-22-07).

PABC was defined as breast carcinoma presenting during the pregnancy and within one year postpartum. Data were extracted from the hospital information system regarding the patient's age at presentation, symptoms, family history, gestation age, clinical stage, histopathology, hormonal receptor status, Ki67, fetal outcomes, disease recurrence, metastasis, and timing of these events. Based on hormonal status, the tumors were classified as triple positive (positive for ER, PR, and HER2); triple negative (negative for ER, PR, and HER2); ER/PR positive, HER2 negative; and HER2 positive, ER/PR negative.

Tumor size (T), lymph node involvement (N), and metastases (M) were assessed as part of TNM staging to classify the clinical stage. Metastatic disease was assessed by the ultrasound of the abdomen, pelvis, and chest X-ray (with a protective abdominal sheet). Treatment given to the patients was also recorded. Data were also collected for disease-free survival and overall survival.

Data were analyzed using IBM SPSS Statistics for Windows, Version 23 (Released 2015; IBM Corp., Armonk, New York). Descriptive statistics were performed, with categorical variables presented as frequencies and percentages. The normality of continuous variables was assessed. As all the continuous variables in our study were non-normally distributed, we used medians and interquartile ranges (IQR) to summarize them. Inferential statistics were performed for patient outcomes. Factors associated with mortality were analyzed using chi-square statistics and the Mann-Whitney U test. Survival analysis for overall survival and disease-free survival was performed using Kaplan-Meier survival analysis. Follow-up duration was used as the time, and mortality was used as the event in the analysis.

## Results

Patient characteristics

The total number of patients was 44, with a median age of 33 years. The median gestational age at the time of diagnosis was 16 weeks. Seven (15.9%) patients had a history of breast carcinoma in their first-degree relatives.

Disease characteristics

The most common presenting symptoms were breast and axillary lumps. Out of 44 patients, 50% (n = 22) presented in the second trimester of pregnancy, 31.8% (n = 14) in the first trimester, and 18.2% (n = 8) in the third trimester.

Intraductal carcinoma (n = 39, 88.6%) is the most common histopathological type. Three patients had intralobular carcinoma (6.8%), whereas one patient presented with metaplastic and another with mucinous carcinoma. Immunohistochemistry revealed that 33 (75%) of the study patients were positive for hormonal receptors, and 11 (25%) were negative for all types of hormonal receptors. Table 1 presents the details of hormonal receptor status. Among the patients with intraductal carcinoma, seven (10.25%) exhibited triple positivity for hormonal receptors, nine patients (23.07%) were triple negative, 20 (51.28%) were ER/PR positive and HER2 negative, whereas three (7.7%) of the ductal carcinoma patients were HER2 positive and ER/PR negative. All three patients with intralobular carcinoma were HER2 positive. At baseline, two patients had metastasis, while seven (15.9%) had advanced carcinoma stages (stages 3 and 4) at the time of diagnosis. Table 1 presents the baseline characteristics of the study population.

**Table 1:** Baseline characteristics of the patients with pregnancy-associated breast carcinoma (PABC) ER: estrogen receptor; PR: progesterone receptor; HER2: human epidermal growth factor receptor 2

Characteristics	n (%)
Age	33 (IQR=30.25-35.75)
Family history	7 (15.9)
Presentation	
Breast/axillary lump	42 (95.5)
Nipple discharge	2 (4.5)
Symptom duration (months)	5 (IQR=3.25-7)
Gestational age at diagnosis (weeks)	16 (IQR=8.25-24)
Hormonal receptor status	
Triple positive	7 (15.9)
Triple negative	11 (25.0)
ER/PR positive, HER2 negative	23 (52.3)
HER2 positive, ER/PR negative	3 (6.8)
Histopathology	
Intraductal	39 (88.6)
Triple positive	7 (10.25)
Triple negative	9 (23.07)
ER/PR positive, HER2 negative	20 (51.28)
HER2 positive, ER/PR negative	3 (7.7)
Intralobular	3 (6.8)
HER2 positive, ER/PR negative	3 (100)
Metaplastic	1 (2.3)
Triple negative	1 (100)
Mucinous	1 (2.3)
Triple positive	1 (100)
Tumor size	
T1	4 (9)
T2	32 (72.72)
T3	6 (13.6)
T4	2 (4.5)
Tumor grade	
2	21 (47.7)
3	23 (52.3)
Nodal involvement	
N0	4 (43.2)
N1	32 (52.3)
N2	6 (2.3)
N3	2 (2.3)
Metastasis	
M0	42 (95.5)
M1	2 (4.5)
Clinical stage	
1	1 (2.3)
2	36 (81.8)
3	5 (11.4)
4	2 (4.5)

Baseline treatment profile of PABC

**Figure 1:**
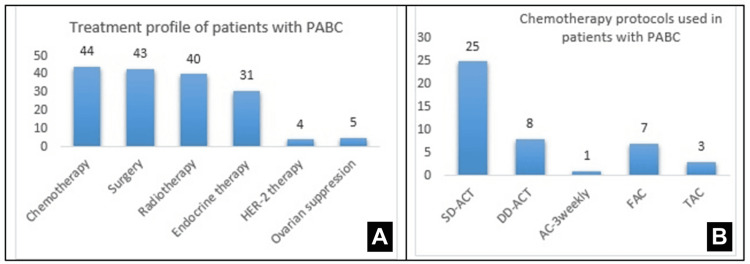
Baseline treatment protocols for patients with pregnancy-associated breast carcinoma (PABC) (A) Treatment profile of patients with PABC; (B) chemotherapy protocols received by the study patients ACT: Adriamycin, cyclophosphamide, Taxol; SD: standard dose; DD: dose-dense; FAC: fluorouracil, Adriamycin, Cytoxan; TAC: docetaxel, Adriamycin, cyclophosphamide; AC: Adriamycin, cyclophosphamide

The median time from diagnosis to the initiation of treatment was 27 weeks (IQR = 8-34 weeks). Chemotherapy was used as a neoadjuvant modality to shrink the tumor size prior to surgery in 32 (72.7%) patients. Chemotherapy was used as adjuvant therapy in 11 (25%) patients and palliative therapy in one (2.3%). Figure 1A shows the treatment profile of patients with PABC. The chemotherapy protocols included a triple therapy with Adriamycin (ADC) and cyclophosphamide (CYC), followed by Taxol at standard dosage (infusions every three weeks) or as a dose-dense regimen (infusions every two weeks) (FAC regimen: Fluorouracil, ADC, and CYC; TAC regimen: Taxotere, ADC, and CYC). A total of 11 (25%) patients underwent upfront surgery. After chemotherapy, a complete pathological response was noted in nine (20.5%) patients, whereas 23 (52.3%) still had residual disease. These patients with residual disease received adjuvant chemotherapy, endocrine therapy, or radiotherapy, depending upon hormonal status. The post-chemotherapy outcome record for one patient was not available. Figure 1B summarizes the details of chemotherapy received by the study patients.

Surgery was performed in 43 (97.8%) patients. One patient was ineligible for surgery due to advanced disease with distant metastasis at the time of diagnosis. She was treated with palliative chemotherapy (AC regimen). Figure 1 summarizes the surgery protocols performed in patients with PABC. We segregated the patients into early (clinical stages 1 and 2) and advanced (clinical stages 3 and 4) breast carcinoma. A total of 37 patients had an early stage of breast carcinoma, where a breast-conserving surgery (BCS) was performed in 18 (48.6%) patients. Thirteen patients underwent BCS with axillary lymph node dissection, while 18 (48.6%) underwent modified radical mastectomy (MRM). Only one (2.7%) of the early breast cancer cases needed a mastectomy. Seven (16.67%) patients had an advanced-stage breast carcinoma at diagnosis. MRM was performed in four (57.1%) patients, while one underwent BCS. Adjuvant radiotherapy was administered to 40 (90.9%) patients following the conclusion of the pregnancy to prevent fetal damage.

Recurrence of the tumor was observed in 22 patients (50%) at a median interval of 31.5 months (range: 20.25-51.5 months) following initial treatment. Among these, four patients experienced a local recurrence, while 17 developed distant metastases. Of the patients with recurrence, 10 underwent surgical treatment, five received a combination of surgery and radiotherapy, and one had both surgery and chemotherapy. Two patients each were managed with radiotherapy or endocrine therapy alone. Notably, two patients were not fit for further treatment. Both patients with baseline metastases experienced recurrence.

Factors associated with mortality

At the end of the follow-up period, the patient outcomes were available for 40 patients. Analyses regarding the factors associated with outcome and survival were performed for these 40 patients. Clinical stage and presence of metastasis at the time of diagnosis did not show a significant association with the likelihood of mortality in PABC. Patients with tumor recurrence had a 23-fold higher likelihood of death. Patients who died had an earlier recurrence of tumors compared to those who survived. Tables 2, 3 show the categorical and continuous factors associated with death in patients with PABC.

**Table 2:** Association of clinical factors with mortality of pregnancy-associated breast carcinoma (PABC)

Clinical Variables	Death	Likelihood Ratio	p-value
Clinical stage at diagnosis			
Early (33)	8 (24.2)	0.94	0.34
Advanced (7)	3 (42.9)		
Recurrence			
Yes (18)	11 (61.1)	22.99	0.000
No (22)	0 (0)		
Metastasis			
Yes (2)	0 (0)	1.33	0.25
No (38)	11 (28.9)		

**Table 3:** Comparison of factors between the outcome groups in pregnancy-associated breast carcinoma (PABC)

Clinical Variables	Alive (Median)	Death (Median)	z-value	p-value
Age at diagnosis (years)	20.17	21.36	-0.29	0.77
Symptom duration (months)	19.66	22.73	-0.76	0.46
Gestational age at diagnosis (weeks)	19.69	22.64	-0.72	0.47
Treatment lag (weeks)	19.41	21.50	-0.52	0.61
Duration to recurrence (months)	23.17	13.45	-2.35	0.02

Fetal outcome and pregnancy management

Pregnancy was concluded before the treatment was started in 12 (28.57%) patients. Out of these, two (16.7%) pregnancies ended in live births, whereas 10 (83.3%) pregnancies ended in spontaneous abortion. All of these patients received neoadjuvant chemotherapy.

Treatment was started during pregnancy in 32 (72.73%) patients. Among these patients, one (3.1%) pregnancy culminated in stillbirth, three (9.4%) in spontaneous abortion, and 27 (84.4%) in live births. Fetal outcome was missing for one (3.1%) case.

Survival analysis

**Figure 2:**
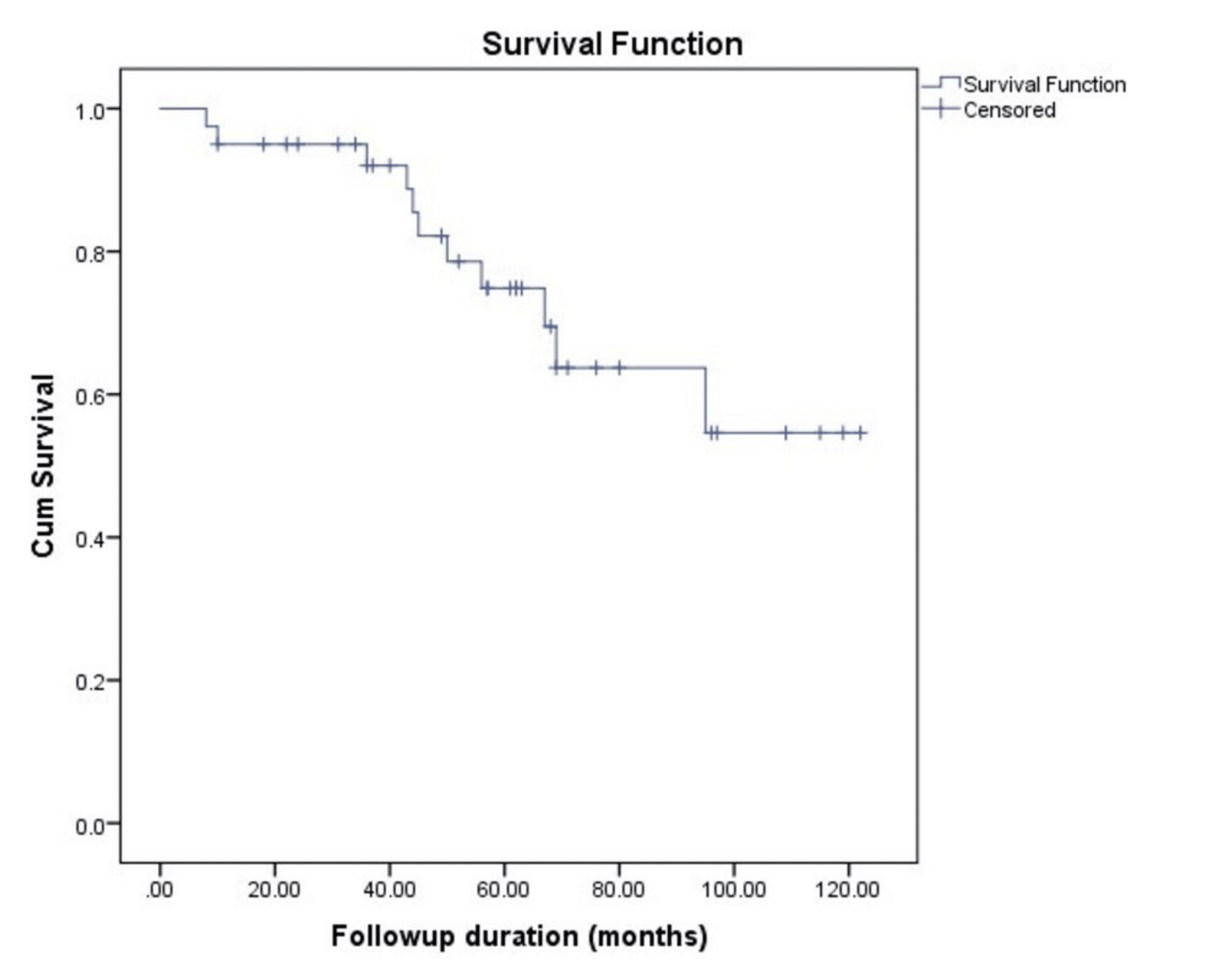
Overall survival of patients with pregnancy-associated breast carcinoma (PABC)

**Figure 3:**
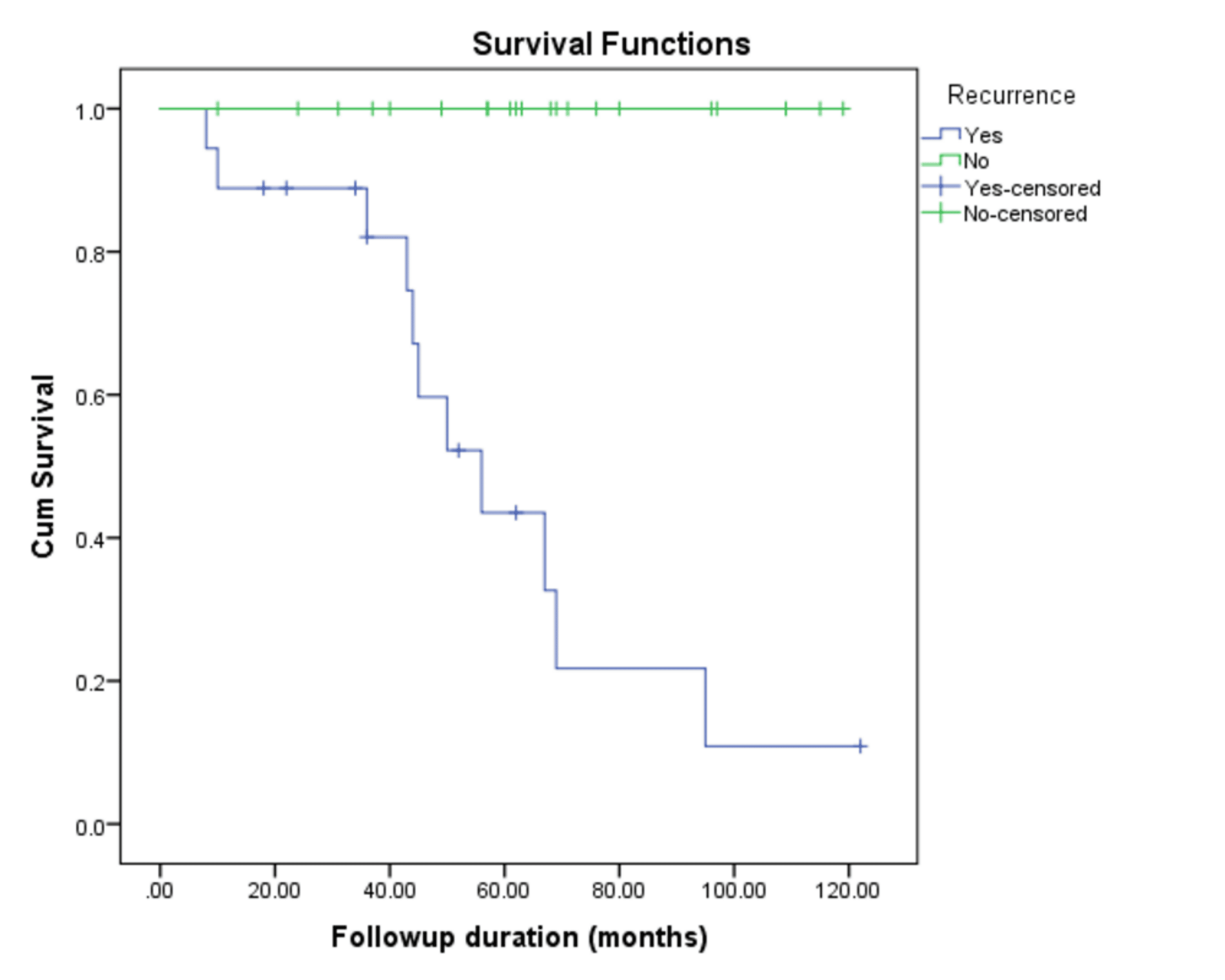
Survival of patients with pregnancy-associated breast carcinoma (PABC) with or without recurrence

The median duration of follow-up in our patients was 56.5 (36.3-69) months. A total of 11 patients died during this follow-up period. The overall mean survival time was 92.6 months (95% CI 78.72-106.47). Out of 18 patients who had the tumor recurrence, 11 (61.11%) died. The median survival was 44.5 (IQR = 31-63.3) months in patients with tumor recurrence, whereas the median survival was 62.5 (IQR = 47.7-84) months in patients who did not have any tumor recurrence (Figures 2, 3).

## Discussion

The incidence of PABC in Pakistan remains inaccurately estimated due to underreporting. This may stem from diagnostic delays due to overlapping symptoms with physiological changes of pregnancy, such as breast engorgement, increased nodularity, skin thickening, and nipple changes, leading to delays in diagnosis, lack of awareness among healthcare providers and patients, and social or cultural stigmas surrounding cancer diagnosis during pregnancy. The combined care of cancer and pregnancy makes it unique in terms of clinical and therapeutic management.

With a median age of 33 years in our study, PABC primarily affects women in their early 30s [9,10]. Pregnancy-related physiological changes may obscure the typical presentation of a breast lump, delaying diagnosis. Improving outcomes and lowering the chance of an advanced-stage diagnosis depend on early identification [11,12]. To determine the most effective treatment approach, understanding the receptor status is crucial [13,14]. The predominance of invasive ductal carcinoma and the variable hormone receptor status in PABC demand a personalized treatment approach [14,15]. Hormone receptor-positive tumors are typically managed with endocrine therapy post-pregnancy, while HER2-positive cancers require targeted anti-HER2 therapies, often delayed to avoid fetal risks. Triple-negative cancers, lacking these targets, rely heavily on chemotherapy, emphasizing the need for timely and tailored interventions based on receptor profiling.

In our study, the majority of tumors were invasive ductal carcinoma (88.6%), and the majority of the tumors were shown to be HR positive by immunohistochemistry (52.3%). This aligns with general breast cancer trends.

It is not recommended to prescribe systemic anti-cancer medications during the first trimester due to their teratogenic effects [16,17].

Anthracycline-based regimens were favored in our PABC patients during the second and third trimesters since they have been demonstrated to be safe without increasing the risk of perinatal fatalities, stillbirths, or long-term cognitive outcomes [18,19].

Limited data exist regarding the safety of taxanes; however, one study has confirmed its safety in PABC. Exposure to taxane chemotherapy may increase the likelihood of neonatal complications, such as admission to the neonatal intensive care unit (NICU) and being small for gestational age [20]. Trastuzumab, tamoxifen, and radiotherapy are known to cause teratogenic impacts antepartum and were effectively utilized in post-pregnancy settings [21].

The high recurrence rate of 50% in PABC highlights the importance of careful follow-up and secondary preventative measures, such as adjuvant therapies, including hormonal therapy, chemotherapy, and HER2 inhibitors, to reduce recurrence. Regular follow-up with imaging and clinical evaluations aids in the early detection of recurrence. Long-term endocrine therapy, such as tamoxifen or aromatase inhibitors, can significantly lower the risk of recurrence in hormone receptor-positive tumors. Notably, patients with recurrence had a 23-fold higher risk of mortality, with recurrence strongly correlating with increased mortality.

Patients who experienced a recurrence had a poorer median survival of 44.5 months compared to those who did not (62.5 months), underscoring the need for early intervention and intensive treatment to lower recurrence rates [21].

The outcomes of pregnancy in PABC depend on the timing of treatment initiation and stage of the disease. High rates of spontaneous abortions were observed in patients who completed pregnancy before treatment, while treatment during pregnancy resulted in higher rates of live births [21]. This diversity highlights the necessity of optimizing both maternal and fetal outcomes through a coordinated strategy combining oncologists, obstetricians, and neonatologists.

Strengths and limitations

This study addresses PABC, an uncommon and underreported illness, especially in low- and middle-income nations where data are limited. The study offers thorough insights into the clinicopathological features, treatment trends, and outcomes of PABC by examining 10 years' worth of data from a tertiary care cancer hospital. Furthermore, the use of strong statistical techniques, such as survival analysis, gives the results more depth and offers insightful data on long-term outcomes. In order to support clinical decision-making, the study also identifies important prognostic markers, including survival, recurrence, and hormone receptor status.

Furthermore, because it is a single-center study, it could not accurately reflect the differences in outcomes and care between various institutions or geographical areas. The absence of long-term follow-up data limits understanding of long-term survival and recurrence trends. The timing of treatment in PABC impacts both maternal and fetal outcomes, with early treatment after the first trimester balancing effective cancer control and reduced fetal risks. Delaying treatment until after delivery may increase disease progression and negatively affect maternal survival.

The research also calls for further studies to refine treatment strategies and evaluate the long-term effects of cancer therapies on both the mother and the fetus.

## Conclusions

PABC is still a major clinical concern, particularly in areas such as Pakistan, where access to healthcare is scarce. Given the particular clinical and therapeutic challenges related to PABC, our study emphasizes the significance of early identification and customized treatment plans. Our cohort's high recurrence rate highlights the necessity of continuous monitoring and the possible advantages of adjuvant treatments in reducing relapse. Future research should focus on improving outcomes for PABC patients by advancing diagnostic methods, such as safer imaging techniques during pregnancy, and refining treatment protocols that balance cancer control with minimal fetal risk. Investigating the long-term effects of treatments and strategies to prevent recurrence is crucial. Additionally, developing sensitive diagnostic tools to detect breast cancer early, tailoring treatment plans based on tumor biology, hormonal receptor status, and patient characteristics, and evaluating the impact of various treatments on fetal health are essential steps. It is also important to establish guidelines to ensure the safety of both mother and child while addressing the emotional and psychological needs of patients and their families through comprehensive care and psychosocial support.
